# Genome Sequences of Five Mycobacterium bovis Strains Isolated from Farmed Animals and Wildlife in Canada

**DOI:** 10.1128/genomeA.00258-18

**Published:** 2018-04-12

**Authors:** Olga Andrievskaia, Marc-Olivier Duceppe, Dara Lloyd

**Affiliations:** a Canadian Food Inspection Agency, Ottawa Laboratory Fallowfield, Ottawa, Ontario, Canada

## Abstract

Mycobacterium bovis is the causative agent of bovine tuberculosis, an infectious disease that affects both animals and humans and thus presents a risk to public health and the livestock industry. Here, we report the genome sequences of five Mycobacterium bovis strains that represent major genotype clusters observed in farmed animals and wildlife in Canada.

## GENOME ANNOUNCEMENT

Bovine tuberculosis (bTb) is a chronic infectious disease caused by Mycobacterium bovis that can affect both animals and humans and therefore presents a risk to public health, the livestock industry, and wild animal species (1). In Canada, bTb has essentially been eliminated from livestock but remains in two wildlife reservoirs, Wood Buffalo National Park (WBNP) and Riding Mountain National Park (RMNP) (2). Whole-genome sequencing was performed on five M. bovis strains representing major genotype clusters observed in Canadian animal farms and wildlife between 1985 and 2015 (3) to provide reference information for epidemiologic investigations of bTb outbreaks and for comparative genomics studies. Strain 2002/0476 was isolated from cattle in the province of Ontario in 2002, strain 2006/0077 was recovered from an elk farm in Ontario in 2006, and strain 2011/0565 was recovered from cattle in British Columbia in 2011. Strain 2008/0665 was isolated from cattle in Manitoba in 2008; this strain type is also associated with a bTb reservoir in wild cervids in RMNP. Strain BMR25/85 was isolated in 1985 from wood bison in WBNP; its genotype represents the WBNP bTb reservoir.

M. bovis isolates genotyped by conventional methods (3) (Table 1) were grown on Middlebrook 7H11 agar for 4 weeks, and genomic DNA was extracted using the MasterPure Gram-positive DNA purification kit (Epicentre, USA). Sequencing libraries for M. bovis 2011/0565 and BMR25/85 were prepared using the Nextera XT DNA library preparation kit (Illumina, Inc., USA) and sequenced on a MiSeq sequencer (Illumina, Inc.) using the MiSeq reagent kit v2 to generate 2 × 250-bp paired-end sequences. The other three strains were sequenced at the Génome Québec Innovation Centre (McGill University, Montreal, Quebec, Canada) with the HiSeq 2000 platform (Illumina, Inc.) to generate 2 × 100-bp paired-end sequences. All five strains were also sequenced using a PacBio RS II sequencer (Pacific Biosciences, USA) at the Génome Québec Innovation Centre.

**TABLE 1:** Genome attributes of M. bovis isolates representing major genotypes observed in Canada between 1985 and 2015

M. bovis isolate	GenBank accession no.	Spoligotype	VNTR^a^ genotype	Genome size (bp)	No. of contigs	Mean coverage (×)
2002/0476	CP027035	SB0265	2-2-3-3-2-2-3-3-2-2-5-3-4-3-4-2-5-3-3-3-2-4-1-2	4,355,637	1	180
2006/0077	PUEG00000000	SB0140	2-2-5-3-2-2-2-3-2-4-6-3-4-5-4-1-5-3-3-3-2-4-1-1	4,343,450	3	246
2011/0565	PUFV00000000	SB0673	2-2-5-3-2-2-2-3-2-4-7-3-4-5-4-2-5-3-3-3-2-4-1-2	4,338,276	2	101
2008/0665	PUEF00000000	SB1071	2-2-5-2-2-2-2-3-2-4-9-3-4-5-4-2-6-3-3-3-2-4-1-2	4,320,841	13	651
BMR25/85	PUEH00000000	SB0130	2-2-5-3-2-2-2-2-2-3-7-3-4-5-4-2-3-3-4-3-2-2-1-2	4,327,606	7	126

a VNTR, variable-number tandem repeat.

Quality trimming and filtering of Illumina reads were performed using the BBTools software suite (http://jgi.doe.gov/data-and-tools/bbtools/). Hybrid assembly of Illumina and PacBio reads was performed with Unicycler v0.4.4 (4), a wrapper tool using minimap v2.5-r622-dirty (5), Racon v0.5.0 (6), SPAdes v3.11.1 (7), and Pilon v1.22 (8). Annotation of the final assemblies was done by the National Center for Biotechnology Information Prokaryotic Genome Annotation Pipeline (9). Assembly metrics are summarized in Table 1. The estimated genome sizes are 4,337,162 ± 13,600 bp (average ± SEM), with a G+C content of 65.61% ± 0.01%, 4,044 ± 23 coding sequences, 45 tRNAs, and 2 clustered regularly interspaced short palindromic repeats (CRISPRs). These values are similar to those of a complete genome of a reference M. bovis strain, AF2122/97 (10).

### Accession number(s).

These whole-genome shotgun projects have been deposited at DDBJ/ENA/GenBank under the accession numbers listed in Table 1. The versions described in this paper are the first versions.
